# Combining statistical analyses and GIS-based approach for modeling the sanitary boundary of drinking water wells in Yaoundé, Cameroon

**DOI:** 10.1016/j.heliyon.2024.e36765

**Published:** 2024-08-23

**Authors:** William Teikeu A, Visiy Edna Buhnyuy, Kasi Njeudjang, Stephane Patrick Assembe, Zakari Aretouyap, Philippe Njandjock Nouck

**Affiliations:** a Department of Environmental Engineering, National Advanced School of Public Works, P.O. Box 510, Yaounde, Cameroon; b Department of Quality Industrial Safety and Environment, Faculty of Mines and Petroleum Industries, University of Maroua, 46, Maroua, Cameroon; c Department of Architecture and Engineering Art, Institute of Fine Arts, University of Dschang, P.O. Box 31, Foumban, Cameroon; d Laboratory of Geophysics and Geoexploration, Department of Physics, Faculty of Sciences, University of Yaoundé I, 812, Yaoundé, Cameroon

**Keywords:** Geographic information system, Groundwater quality, Multivariate analyses, Pit latrines, Sanitary boundary, Wells

## Abstract

In slums and urban areas with unplanned housing such as in the city of Yaounde, Cameroon, poor water quality and inadequate sanitation pose significant health risks. The absence of locally-defined sanitary boundaries, tailored to hydrogeological conditions, hinders effective zoning and land use planning, exacerbating environmental degradation and health hazards. In this study, the sanitary boundary between drinking water wells and sources of pollution in the city of Yaoundé was defined using statistical analysis techniques and Geographic Information Systems (GIS). The Groundwater Quality Index (GWQI) and certain significant parameters affecting water quality notably the transmissivity of the aquifer and well depth were used to establish the sanitary boundary equation which was then interpolated in a GIS environment to obtain the sanitary boundary map of the study area. The linear equation deduced from the significant factors was defined to map the health boundaries between wells and pollution sources for a nominal value of the GWQI (GWQI = 25). Of the 112 wells analysed, 37 % had an excellent GWQI, 16 % were good while the remaining 47 % were poor. Statistical analysis showed a strong correlation between the GWQI and significant factors of groundwater pollution, such as the distance between well and pit latrines (r = −0.753), the aquifer transmissivity of the formation (r = 0.671) and the depth of the wells (r = - 0.855) but no correlation with elevation (r = 0.017) and well age (r = 0.090). Linear regression analysis confirmed the association of the GWQI with the main factors of pollution (p ≤ 0.05). A coefficient of determination of R^2^ = 0.85 was obtained when validating the linear regression plot based on independent data between measured and predicted GWQI. The sanitary boundary map shows that the wells in our study area should be located between 39 m and 370 m, with an average value of 215 m. New regulations on the distance between well and pit latrines are essential to prevent groundwater pollution.

## Graphical abstract

**Figure:**
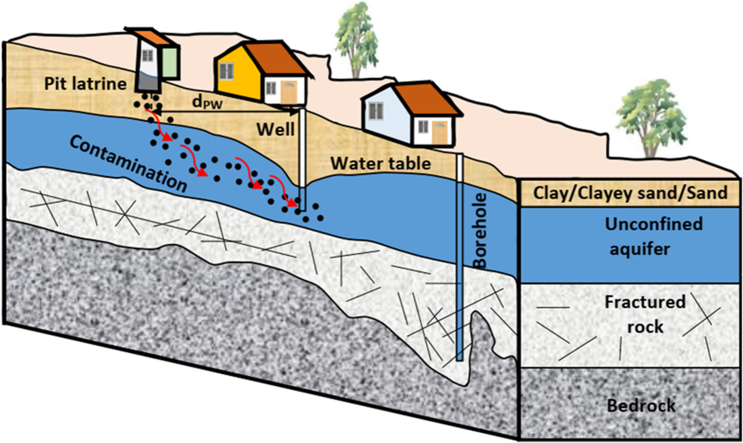

## 1 Introduction

Over the past few decades, the advent of on-site sanitation has significantly improved the lives of millions of people especially in developing countries [1]. However, improved sanitation has also become a threat to groundwater resources in Sub-Saharan Africa, particularly in regions where rainfall is almost non-existent for several months of the year [[2], [3], [4], [5]]. Urban expansion and population growth have been accompanied by the use of pit latrines, which are often poorly constructed and built close to wells due to lack of space leading to groundwater contamination [1,6,7]. Gwenzi et al. [8] pointed out that contaminants present in pit latrines can migrate to surface and groundwater, which serve as sources of drinking water, posing a threat to human health. In addition, Elemile et al. [9] observed that there is significant regional heterogeneity in the impacts of abattoir operations on groundwater quality which is largely due to the percolation of abattoir effluent into the ground, as seen by the groundwater contamination inventory map. The contamination of drinking water wells by faecal bacteria is among the leading causes of diarrheal diseases in developing countries [10,11] with about 1.8 million people in Africa dying annually from diarrhea; 80 % of whom are children under five years [12]. However, Ravenscroft et al. [13] intimates that the expansion of latrine coverage should not be impeded by fears of contaminating groundwater.

The suitability of drinking water for human consumption is typically assessed using the Weighted Arithmetic Water Quality Index. This method has been used widely in water quality assessment and management across the world [[14], [15], [16]]. It is based on varying numbers and types of water quality parameters and summarizes large amounts of quality data into simple terms (excellent, good, poor, very poor and unsuitable) based on their obtained numerical value [15]. For groundwater, many researchers have developed a specific index, technically known as the Groundwater Quality Index (GWQI) [14,17,18]. It is one of the most effective tools for providing comprehensive information on groundwater quality based on a group of hydrogeochemical parameters [18]. In general, the GWQI is a practical and relatively simple approach for assessing the impact of overall groundwater pollution, even though it can be challenging to identify the sources of pollution [19]. Statistical methods involving the GWQI and Geographic Information System (GIS) tools have been used in several countries to prioritize better management of sanitation and water resources. Hossain et al. [14] presented a groundwater quality estimation model in Sharsa Upazila area, Jashore district, Bangladesh using multivariate statistics and geographic information system techniques. They estimated groundwater quality assessment using GWQI and Wilcox plot with acceptable accuracy at all sampling sites. Shahsavani et al. [20] investigated the chemical quality of drinking water in Shiraz, Iran through a new water quality index designed by fuzzy multi-criteria group decision-making methods, merged with GIS. Azage et al. [21] used spatial analysis to demonstrate that there are geographical variations and inequalities in access to drinking water and sanitation in Ethiopia.

The contamination of well water by microorganisms and chemicals from sanitation facilities is caused by several factors notably; the proximity of latrines to the well, soil type, well depth and elevation [[22], [23], [24], [25]]. Shallow wells are more likely to get contaminated compared to deep wells [24,25]. In addition, wells located in low relief areas are more likely to be contaminated with faecal matter and are a greater source of diarrhea outbreaks, compared to wells situated in areas with high elevation [26]. Chaúque et al. [27] studied the spatial arrangement of wells and latrines and their influences on water quality in a clay soil in Mozambique and found that the clay soil appeared to mitigate the expected high levels of microbial contamination. Elisante et al. [24] observed that water sources that were located within 10 m of pit latrines had the highest coliform counts relative to those located beyond 10 m. Guidelines exists for the minimum distance between water wells and latrines, varying from 10 to 75 m internationally [7]. While a minimum distance of 15m is recommended by the World Health Organisation (WHO), Ngasala et al. [25] showed that site-specific distances are required to separate domestic wells from pit latrines due to variations in hydrogeological conditions. The actual distance is greatly affected by the aquifer properties which influences the rate of transportation of microbiological and chemical contaminants [25]. It is therefore necessary that experiments be conducted in various locations to model the sanitary boundary using local conditions.

In Cameroon, standards for pit latrines and their location in relation to wells are not applied, particularly in urban and peri-urban areas. In the unplanned urban areas of Yaoundé, where the vast majority of people are poor, wastewater management is difficult due to poor access to roads and the lack of sewerage systems. Pit latrines are poorly constructed, leading to pollution of domestic wells [28]. Improving sanitation is an absolute necessity, given the epidemic and even pandemic risks affecting the city of Yaoundé and Cameroon in general [29]. While previous studies have investigated specific aspects of environmental pollution in Yaoundé including water contamination of groundwater by latrines [[30], [31], [32], [33]], a thorough examination of the sanitary boundary remains a significant knowledge gap. Notably, Djousse et al. [28] conducted a study in the Melen slum in Yaounde, employing simple linear regression to estimate the minimum distance required between pit latrines and groundwater sources. They recommended a minimum distance of 42.89 ± 16.76 m but their study was limited in sampling size as only 19 wells were analysed. The absence of a clear sanitary boundary based on the local hydrogeological conditions compromises effective land use planning and zoning exacerbating environmental pollution and health risks. This research seeks to fill this knowledge gap by establishing Yaoundé’s sanitary boundary using a combination of statistical and GIS tools, providing crucial insights for informed policy-making.

## 2 Materials and methods

### 2.1 Description of the study area

**Fig. 1:**
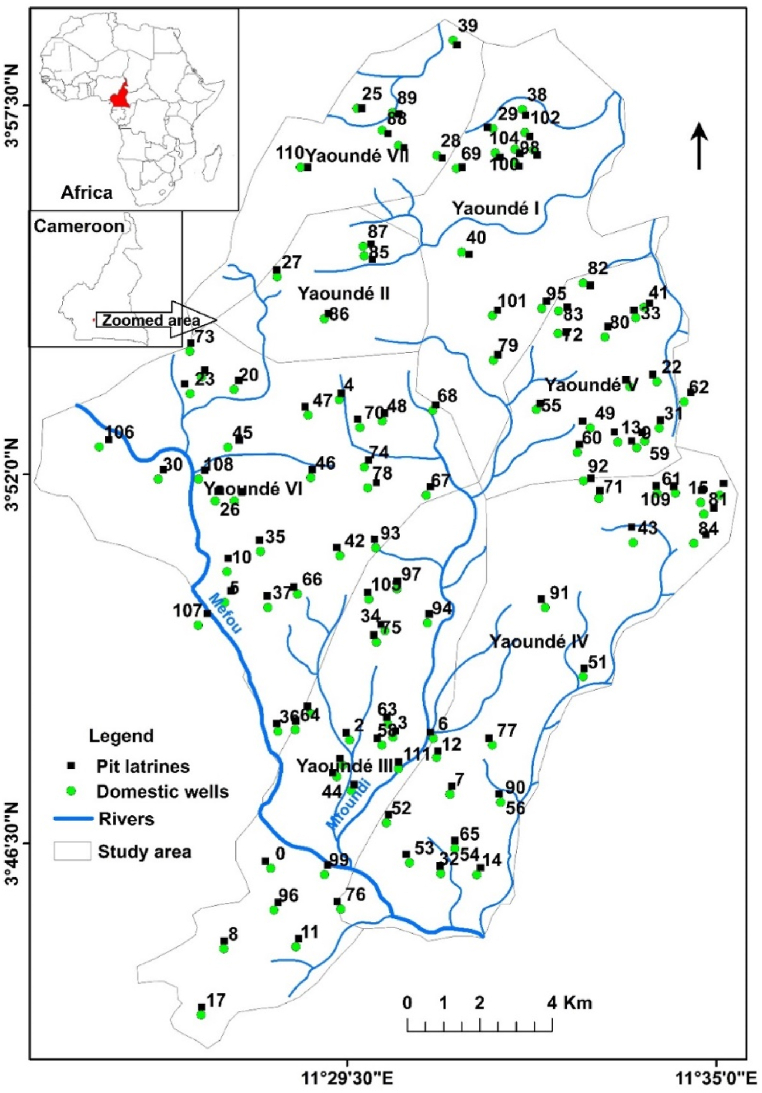
The location of the study area (Yaoundé), in Cameroon, Africa; showing the wells and latrines used for the study.

The study was conducted in the city of Yaoundé (Fig. 1) located between latitudes 3° and 5° north and between longitudes 11° and 13° east and is divided into seven districts (Yaoundé I-VII). This is a vast, gently undulating equatorial region with a thick lateritic, sandy and clay soil developed on a Precambrian bedrock [34]. The region is a plateau, 600–800m above sea level, deeply eroded by a very active hydrological network, with the main rivers being Mefou and Mfoundi [35]. This drainage has created shallow valleys of varying widths (from 10m to 75m) and the resulting relief includes mountain ranges, the Yaoundé plateau and low marshy valleys [36,37]. There are two rainy seasons, from August to November and from March to May; and two dry seasons, from December to February and from June to August. The average rainfall is 1626 mm/year and the average daily temperature is 24.17 °C [38]. The population density of the study area is approximately 9095 inhabitants/km^2^ [39]. The region has a wide range of social classes, from affluent to low-income families. The houses are very close together and very few are connected to the sewerage system [40].

The study area consists mainly of two aquifers: an unconfined upper clay, clayey-sandy and sandy aquifer and a confined lower fractured granite-gneiss aquifer [41]. The region contains over 250 wells with a majority of wells in this area drawing water from the unconfined aquifer [42]. The average depth of the water table is several tens of metres below the surface. Most of the wells are owned by private individuals, but they are considered public wells as they are used daily by the surrounding population [43,44]. Water is generally pumped from these wells in the morning and evening for between 4 and 6 h a day. The city principally makes use of onsite sanitation systems notably pit latrines and septic tanks which are most often poorly designed/constructed with little or no consideration for human and environmental health, affecting access to water and sanitation [45].

### 2.2 Data collection

Of the approximately 250 drinking water wells in the study area, 112 wells were selected on the basis of the qualitative information available. The location of the wells and latrines considered in this study is presented in Fig. 1. All the wells were hand dug, cylindrical in shape and without internal lining. Official reports on the wells were collected from the Ministry of Water and Energy in Cameroon and included information such as the date of drilling, the physico-chemical parameters, the elevation from sea level, the transmissivity of the aquifer formation, and the depth of the well. For each well, the GPS location (latitude and longitude) was recorded as well as the distance between the well and the nearest pit latrine. To assess water quality, water samples were collected from each well during the dry season (June–August 2015 and February–March 2017) and analysed using standard methods [46]. Eleven water quality parameters were selected including: calcium (Ca^2+^), Sodium (Na^+^), Magnesium (Mg^2+^), Potassium (K^+^), Iron (Fe^2+^), Carbonate (${HCO}_{3}^{-}$), Chloride (Cl^−^), pH, Electrical Conductivity (EC), Total Dissolved Solids (TDS) and Total Hardness (TH). These parameters were then used to estimate the GWQI. However, the determination of GWQI was greatly limited by the lack of microbiological parameters.

### 2.3 Water quality analysis

The water quality of the wells was determined using the GWQI. WHO standards (Table 1) were used to calculate hydrogeochemical parameters which were then used to estimate the GWQI for the different water samples. The calculation of the GWQI was completed in three stages.

**Table 1:** Parameters and their relative weights used for the calculation of the GWQI according to WHO [47].

Measurement Variable	EC	Na^+^	K^+^	TH	Fe^2+^	TDS	pH	Cl^−^	${HCO}_{3}^{-}$	Mg^2+^	Ca^2+^
Measurement Unit	μs/cm	mg/l	Mg/l	mg/l	mg/l	mg/l	–	mg/l	mg/l	mg/l	mg/l
Quality Guidelines	300	200	12	35	0.3	500	6.5–8.5	200	240	30	75
Relative weighting	0.0009	0.0012	0.0230	0.0079	0.920	0.0006	0.0345	0.0014	0.0012	0.0092	0.0037

EC = Electrical Conductivity; Na^+^ = sodium; K^+^ = Potassium; TH = Total hardness; Fe^2+^ = Iron; TDS = Total Dissolved Solids; pH = Hydrogen potential; Cl^−^= Chloride; HCO_3_^−^ = Carbonate; Mg^2+^ = Magnesium; Ca^2+^ = Calcium.

First, a weight was assigned to the measured parameters and the relative weight calculated using Eq. (1).

(1) Wi=wi∑i=1nwi

where w_i_ is the weight of each parameter and n is the total number of parameters measured.

Second, the quality assessment scale for each parameter was calculated using Eq. (2) and following their respective standards as outlined in Ref. [47].

(2) qi=(CiSi)×100

where *qi* is the quality rating, *Ci* is the concentration of each parameter and *Si* is the standard value of each parameter [47].

Finally, the sub-indices for each parameter were then calculated using Eq. (3) and then summed to give the GWQI for each groundwater sample Eq. (4).

(3) $${SI}_{i}=W_{i}\times q_{i}$$

(4) GWQI=∑i=1nSIi=∑i=1n(Wi×qi)=∑i=1n(wi∑i=1nwi×(CiSi)×100)

where SI_*i*_ is the sub-indices of *i*th parameter, *qi* ranking according to the concentration of *i*th parameter, and *n* the number of parameters.

### 2.4 Modelling the sanitary boundary using statistical techniques

The sanitary boundary was modelled by determining the equation of best fit between ground water quality (measured in terms of the GWQI) and the factors affecting the quality of water in the wells (distance between well and pit latrines, elevation, transmissivity of the aquifer formation, well age and depth). In order to decipher the relationship between the GWQI and these factors, the data was subjected to two statistical analysis methods, namely 1) Multivariate correlation to determine the empirical relationship between the GWQI and the independent variables, and 2) Linear regression to confirm whether there were significant linear relationships between the GWQI and the parameters affecting the wells.

For the regression analysis, a general linear equation was fitted to the GWQI values as a function of the parameters affecting the wells. All possible combinations of equation parameters were evaluated to identify the expression that best explained the data based on the coefficient of determination (R^2^) and p-value. The best equation was selected using two techniques; the progressive method and the data collinearity. The collinearity occurs when one predictor variable in a model can be predicted linearly from the others [48]. High collinearity between predictor variables indicates that the variables share a significant amount of information [49]. This can be a problem when estimating parameters for any descriptive dataset, as it increases the variance of the regression parameters, which can lead to misidentification of the predictors [50,51]. Eqs (5), (6) describe the general form of the regressions considered.

(5) $$Y=\beta_{0}+\beta_{1}x_{1}+\beta_{2}x_{2}+\beta_{3}x_{3}+\beta_{4}x_{4}+\beta_{5}x_{5}+\varepsilon$$

(6) $$Y=\beta_{0}+\beta_{1}\left(T_{r}\right)+\beta_{2}\left(W_{depth}\right)+\beta_{3}\left(L_{pw}\right)+\beta_{4}\left(Elevation\right)+\beta_{5}\left(Age\right)$$

where Y is the dependent variable (GWQI); *β*_*1-5*_ is the linear regression coefficients; *β*_*0*_ is an intercept; *x*_*1-5*_ is the independent variables (Factors affecting groundwater quality); *ε* unexplained error variance; T*r* is the transmissivity of the aquifer formation; W_*depth*_ is the well depth; and *Lpw* is the distance between pit and well.

Of the 112 drinking water supply wells sampled, 80 % of the well data was used to develop the linear regression expression and the remaining 20 % of the data was used to validate the expression. The linear regression equation was obtained using the stepwise method so that parameters that do not have a significant relationship with the GWQI are eliminated from the final equation. The linear regression equation for the GWQI was validated by the coefficient of determination. The sanitary boundary (minimum distance between pit and well) was then defined on the basis of the significant parameters affecting groundwater quality and the standard average value of the groundwater quality index (GWQI = 25), which guarantees drinking water of very good quality. The GWQI values are generally classified into five categories: excellent (GWQI < 50), good (50 < GWQI < 100), low (100 < GWQI < 200), very low (200 < GWQI < 300) and unsuitable for consumption (GWQI >300) [15,16]. In other words, the GWQI is one of the input data to the final regression expression and the parameter of interest is the sanitary boundary. All statistical analyses were conducted using the IBM Statistical Package for the Social Science (SPSS) version 24.0.

### 2.5 Production of the sanitary boundary map using GIS

**Fig. 2:**
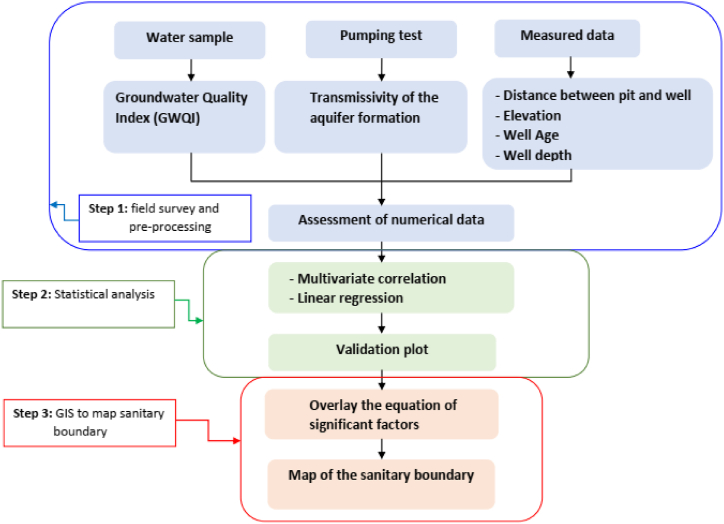
The flow chart showing the methodology stages used in this study.

GIS helps to statistically interpolate different experimental data to produce thematic layers and spatial maps [51]. It limits the uncertainty factors of the groundwater hydraulic parameters and allows the relationship to be established in a statistical approach to summarise the quality of the groundwater in the study area in a simple image format [52]. Inverse distance weighting is the most widely used and popular method for generating spatial distribution maps [53]. In this study, GIS was used as an effective tool to provide digital maps and to model the sanitary boundary of the study area. As the data for this study was less dense, various thematic maps (for example well depth and transmissivity of the aquifer formation) were provided by the Nationale Institute of Cartography of Cameroun. These two digital maps were generated in the GIS environment using the inverse distance weighting (IDW) interpolation method. In our study, the thematic maps were overlayed to run the model and define the health boundaries of the entire region at a grid resolution of 50m × 50m. The steps of the procedure can be summarised in a flow chart as shown in Fig. 2.

## 3 Results

### 3.1 Groundwater quality analysis

**Fig. 3:**
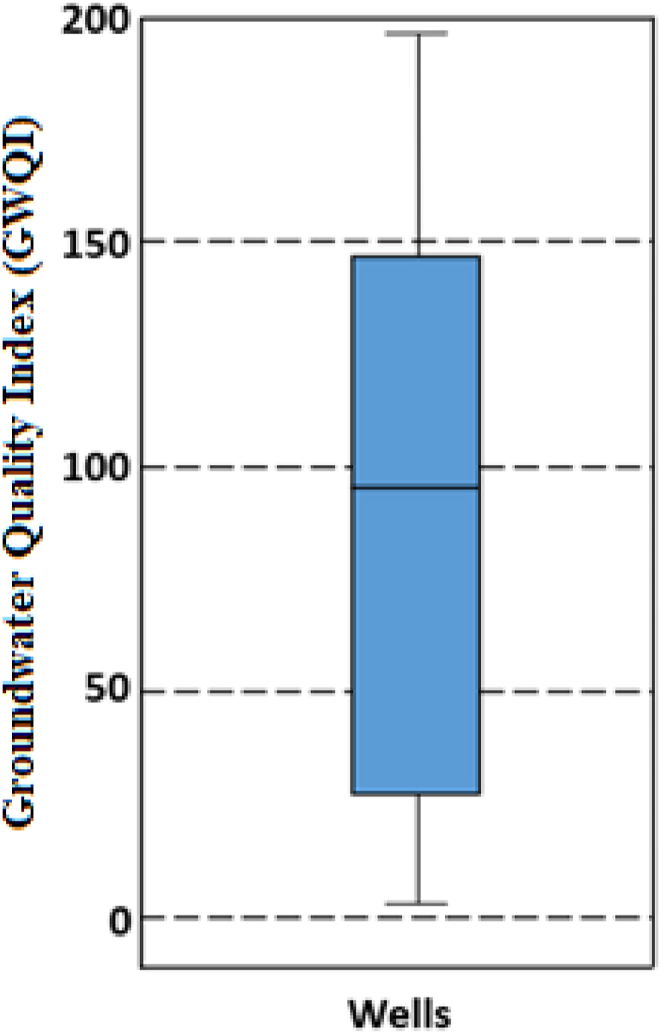
The box plots showing the upper adjacent, median and lower adjacent values of the groundwater quality index (GWQI) of the sampled wells.

Water quality was assessed using the weighted arithmetic groundwater quality index. The water with a GWQI of less than 50 is considered to be of excellent quality, while values between 50 and 100 indicate water of good quality. The GWQI values between 100 and 200 indicate water of poor quality, while values above 200 indicate water of very poor quality, unfit for consumption. The water samples collected, analysed and interpreted from the 112 domestic wells had a GWQI ranging from 2.76 to 196.24, with an average of 91.27 (Fig. 3). 37 % of these wells had an excellent GWQI, 16 % were good and the remaining 47 % were poor.

### 3.2 Multivariate correlation

Several correlative comparison scenarios were run to determine the statistical significance of the explanatory variable (e.g. GWQI) on distance between well and pit latrines, well age, elevation, transmissivity of the aquifer formation, and well depth across all sampling locations. The GWQI was a statistically significant predictor of distance between well and pit latrines (r = - 0.753), well depth (r = - 0.855) and transmissivity of the aquifer formation (r = 0.671). The combinations GWQI - elevation and GWQI - well age gave correlation coefficient values of 0.017 and 0.090 respectively, suggesting that there is no correlation between water quality and these parameters. The GWQI was directly related to the transmissivity of the aquifer formation and inversely related to well depth and the distance between wells and pit latrines. Well depth had a significant direct correlation with the distance between well and pit latrines (r = 0.688) and an inverse correlation with the transmissivity of the aquifer formation (r = - 0.626). There was no significant correlation between the elevation and depth of the well (r = 0.062), the distance between well and pit latrines (r = −0.021), and the transmissivity of the aquifer formation (r = −0.065). No significant correlation was obtained between the age of the well and the depth of the well (r = −0.047), the distance between well and pit latrines (r = −0.145), and the transmissivity of the aquifer formation (r = 0.037). Finally, there was no significant correlation between elevation and well age (r = −0.020). Based on these results, the most important factors in the GWQI are the distance between well and pit latrines, the transmissivity of the aquifer formation and the depth of the well. The parameters affecting groundwater quality and the GWQI values for certain wells are shown in Table 2. The results of the correlation analysis of the GWQI with the significant factors are presented in Table 3.

**Table 2:** Characteristics of the 28 randomly sampled wells used in the validation stage of the regression model.

No.	Longitude (°)	Latitude (°)	actual GWQI	Elevation (m)	Distance from LW (m)	Well depth (m)	Transmissivity (m^2^/day)	Well age (Year)	Predicted GWQI
0	11.472710	3.768865	172.124	575.000	27.000	19.000	7.903	19	132.738
11	11.478943	3.749364	143.009	702.000	50.000	12.590	5.909	16	130.723
15	11.579444	3.859722	59.761	698.000	76.000	31.010	2.249	23	63.405
16	11.584204	3.861545	2.758	711.000	111.000	39.480	3.186	22	33.400
21	11.455501	3.890899	20.816	732.000	105.000	28.000	1.874	19	57.370
22	11.568570	3.889760	9.559	696.000	129.000	43.280	0.521	18	7.071
26	11.458870	3.860100	140.914	753.000	40.000	11.580	0.328	15	115.443
27	11.474290	3.915780	76.142	854.000	84.000	35.220	3.661	13	55.904
38	11.535167	3.957377	33.346	679.000	90.000	35.860	2.457	13	47.362
49	11.552083	3.878250	93.458	749.000	69.000	34.100	1.717	16	56.961
51	11.550278	3.816457	79.050	698.000	63.000	38.220	3.422	9	56.392
53	11.507167	3.770233	139.696	712.000	3.000	10.730	4.838	11	149.649
54	11.518417	3.773733	184.465	707.000	22.000	4.280	6.657	11	164.090
55	11.538644	3.882864	16.123	703.000	83.000	23.290	1.440	12	75.401
56	11.529806	3.785278	121.251	706.000	7.000	11.060	2.264	12	137.316
57	11.490556	3.794444	137.932	674.000	12.000	12.000	2.608	13	134.471
65	11.518417	3.773733	163.701	685.000	10.000	17.970	6.484	15	136.418
70	11.494817	3.878350	180.775	736.000	8.000	4.540	5.302	17	163.824
73	11.452600	3.897300	8.542	761.000	116.000	48.480	0.750	17	1.074
78	11.496758	3.863361	156.580	753.000	10.000	9.000	1.841	19	139.263
90	11.529806	3.785278	10.361	705.000	21.000	37.050	0.655	24	65.149
91	11.540843	3.833605	196.235	754.000	8.000	3.990	5.728	24	166.751
95	11.539992	3.907944	139.107	755.000	95.000	12.860	6.070	26	112.767
99	11.486053	3.767282	151.658	696.000	11.000	8.320	2.946	27	144.725
101	11.527756	3.906179	68.773	750.000	62.000	50.310	5.331	29	36.111
108	11.454750	3.865510	84.815	711.000	58.000	27.220	2.045	33	78.597
109	11.573167	3.862056	160.130	638.000	25.000	15.330	6.875	33	138.078
110	11.480087	3.942989	145.460	924.000	8.000	17.730	4.184	40	128.864

GWQI = Groundwater Quality Index; LW = Latrine to Well.

**Table 3:** Multivariate correlation analysis between groundwater quality index and the effective factors.

Independent variable	Correlation coefficient					
	GWQI	Elevation	Distance between well and pit	Well depth	Transmissivity of aquifer	Well age
GWQI	1					
Elevation	0.017	1				
Distance between well and pit	−0.753	−0.021	1			
Well depth	−0.855	0.062	0.688	1		
Transmissivity of aquifer	0.671	−0.065	−0.464	−0.626	1	
Well age	0.090	−0.020	−0.145	−0.047	0.037	1

GWQI = Groundwater Quality Index.

### 3.3 Linear regression

The regression analysis indicated that the transmissivity of the aquifer formation, the depth of the well and the distance between the well and the pit latrine were the most significant factors in establishing the sanitary boundary. The linear regression expression was obtained using the stepwise method. The effectiveness of the linear regression was assessed by applying it to wells whose data were not used to define the equation. The combinations of GWQI - well depth and GWQI - distance between well and pit latrine showed a significant difference with a p-value of 0.000 for each. The combination of GWQI and transmissivity of the aquifer formation also showed a significant difference with a p-value of 0.050. Table 4 summarizes the results of the multiple linear regression for the combination of independent variables tested with the GWQI. For the t-statistic, the combinations of GWQI with well depth, distance between well and pit latrines and transmissivity of the aquifer formation gave values of −6,709, −4442 and 1985 respectively. The standard errors for the same combination were 0.348, 0.090 and 1.951 respectively.

**Table 4:** General linear model analysis for GWQI (linear model, stepwise method, R^2^ = 0.796).

Parameter	Coefficient (β)	Standard error	t statistic	p-value
Intercept	157.022	12.815	12.253	0.000
Well depth (m)	−2.332	0.348	−6.709	0.000
Distance between well and pit (m)	−0.399	0.090	−4.442	0.000
Transmissivity of aquifer (m^2^/day)	3.873	1.951	1.985	0.050

The linear regression expression which best suited the data is given in Eq. (7).

(7) $$GWQI=3.873T_{r}-2.332W_{depth}-0.399L_{pw}+157.022$$

where GWQI is the groundwater quality index, *Tr* is the transmissivity of the aquifer formation (m^2^/day), W_*depth*_ is the depth of the well (m), and Lpw is the distance between the pit and well (m) also known as the sanitary boundary.

**Fig. 4:**
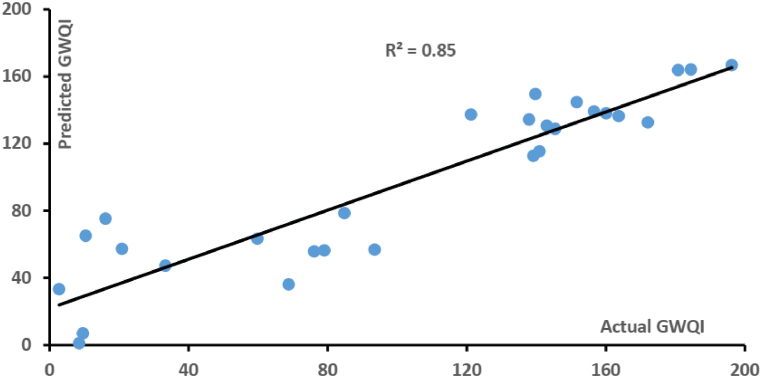
The comparison between predicted and actual GWQI values in the validation stage.

The validation plot for the linear regression expression between the observed and predicted values of the GWQI is shown in Fig. 4. The coefficient of determination is significant (R^2^ = 0.85).

Rearranging Eq. (7) leads to Eq. (8). Dividing all the coefficients in Eq. (8) by 0.399 gives the sanitary boundary (Eq. (9)).

(8) $$0.399L_{pw}=3.873T_{r}-2.332W_{depth}-GWQI+157.022$$

(9) $$L_{pw}=9.707T_{r}-5.845W_{depth}-2.506GWQI+393.539$$

Given that the GWQI < 50 corresponds to the excellent water quality index [15,16], and considering an average standard value of the GWQI = 25 in Eq. (9), we obtained the final expression, which depends only on the transmissivity of the aquifer formations and the depth of the well, and allows us to define the sanitary boundary (Eq. (10)). This expression can be used to express the sanitary boundary for future wells if the transmissivity and depth of well is known.

(10) $$L_{pw}=9.707T_{r}-5.845W_{depth}+330.889$$

### 3.4 Sanitary boundary map

**Fig. 5:**
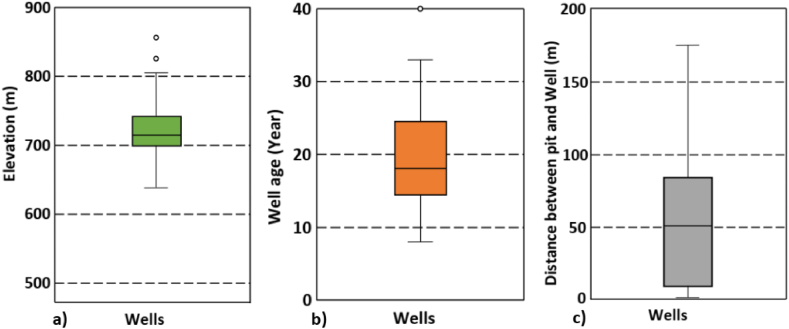
The box plots showing **a)** the upper adjacent, median and lower adjacent values of elevation, **b)** well age, and **c)** distance between well and nearest pit latrine.

Fig. 5 shows the boxplot of elevation, age of wells and distance between well and pit latrines in the study area. The elevation of the wells varied between 575 m and 981 m above sea level, with an average of 725.77 m (Fig. 5a). The age of most wells at the time of sampling ranged from 8 to 43 years, with an average of about 19 years (Fig. 5b), and with eight wells being over 30 years old. Field measurements showed that 32 % of the wells were less than 15m from pit latrines. The minimum distance was 1 m and the maximum 175 m, with an average of 53.60 m (Fig. 5c).

**Fig. 6:**
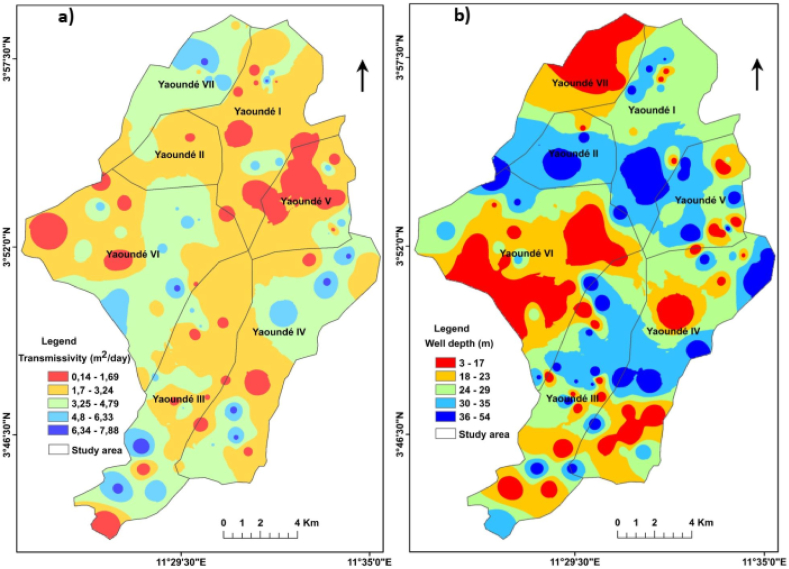
The spatial distribution of a) the aquifer transmissivity and b) wells' depth within the study area.

The spatial mapping of the transmissivity of the aquifer formation and the depth of the wells in the study area is shown in Fig. 6. The transmissivity of the aquifer formation varied between 0.14 and 7.88 m^2^/day with low values observed in the western, central and north-eastern parts of the region (Fig. 6a). The highest values were recorded in the south, east, south-west and extreme north-west of the region. The spatial map for well depth varied between 3 and 54 m and shows low values in the western, northwestern and southern parts of the study area (Fig. 6b). The highest values are found in the northern, eastern and south-eastern parts of the study area. Among other things, there are alternating high and low groundwater depths in the central part of the study area.

**Fig. 7:**
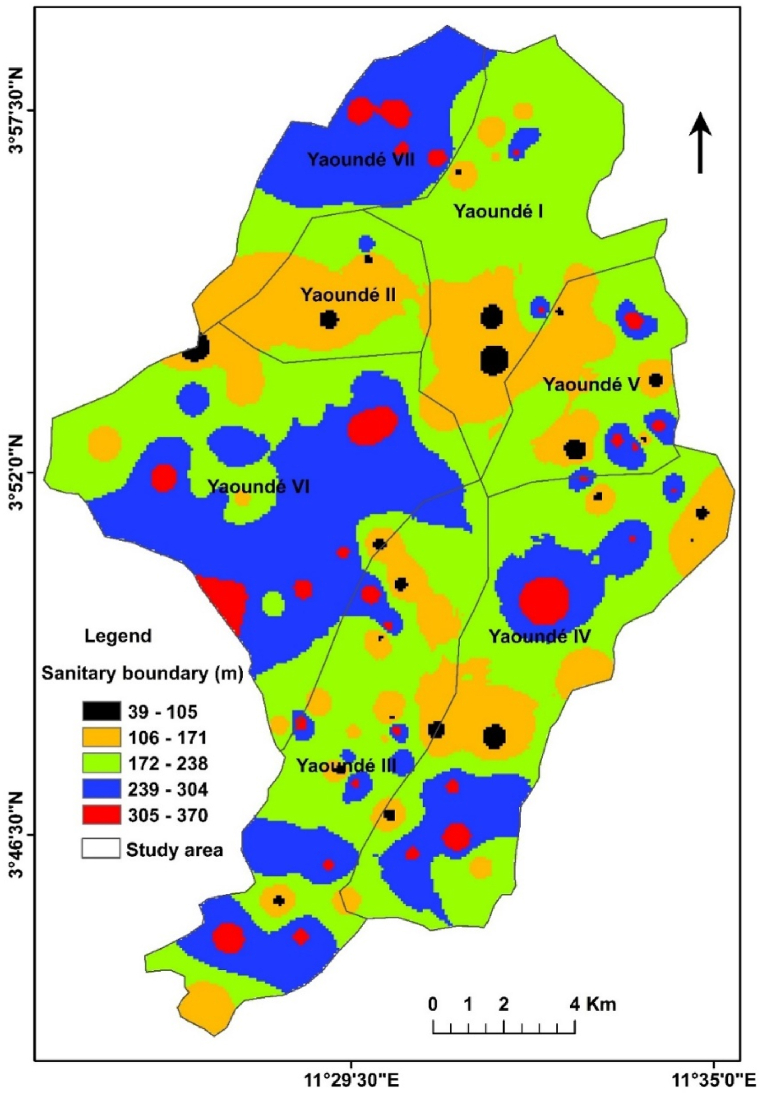
The map of sanitary boundary of the drinking wells for ensuring proper drinking water quality.

The sanitary boundary map as shown in Fig. 7, obtained by adding Eq (9) in a GIS environment, varied between 39 and 370 m with an average value of 215 m. Overall, the sanitary map is divided into 5 sanitary boundary ranges (R1 to R5). R1 varies from 39 to 105 m, R2 from 106 to 171m, R3 from 172 to 238 m, R4 from 239 to 304 m and R5 from 305 to 370 m. A combination of various ranges was observed in the different districts of Yaoundé. In Yaoundé I, the northern part was dominated by R3 with a slight intrusion of R2, R4 and R5 respectively while the southern part had more of R2 with slight intrusion of R1, R3 and R5. In Yaoundé II, the western, central and eastern sections had the R2 sanitary boundary range while the southern and northern sections had the R3. However, spots of R1 and R4 could also be observed. Yaoundé III had a very diverse sanitary boundary comprising all the ranges with the principal range being R3 followed by R4. Yaoundé IV was equally very diverse with R3, R4 and R2 being more prominent. In Yaoundé V, all ranges were present with R3, R2 and R4 being more pronounced respectively. In Yaounde VI, the R4 and R3 ranges dominated with slight intrusions of R2, and R5. In Yaounde VII, the northern part was dominated by R4 with slight intrusions of R5 while the southern part mainly had the R3 and R2 ranges. In general, the R3 (172–238 m) was the most occurrent followed by R4 (239–304 m) and R2 (106–171). The extremes R1 (39–105) and R5 (305–370) could only be observed in spotted areas throughout the study area.

## 4 Discussion

The physico-chemical characterisation of water is fundamental to the sustainable management of water resources. Having reliable data guides the actions that need to be taken to manage water resources effectively [54]. In Cameroon, water quality is still insufficiently considered by water supply project developers, who are more concerned with the financial aspects. The study was conducted in an urban area with no observed agricultural or landfill sites. The only sources of groundwater pollution should therefore be organic matter, such as human waste from pit latrines and domestic activities carried out close to the wells. Field surveys showed that the water in most of the wells had an unpleasant taste with high Fe content. This was not surprising given that the average value of the water quality index for all 112 wells sampled was 91.27 indicating that the water was not of excellent quality. As the study area is highly altered and ferruginous, a high concentration of iron in drinking water creates an unpleasant metallic taste [55]. Khatri et al. [56] summarised the different approaches used worldwide to remove high levels of iron from water, namely: conventional strategies, biological strategies, strategies based on membrane technology, and strategies based on nanotechnology.

Statistical analysis of the GWQI with factors affecting groundwater quality showed a correlation with the transmissivity of the aquifer formation, well depth and distance between well and pit latrines, but no correlation with elevation and age, suggesting that elevation and age of well do not affect groundwater quality. These results are consistent with the work of Gholami et al. [57] on the southern coasts of the Caspian Sea, Iran, who conducted a statistical analysis and found that the transmissivity of the aquifer formation, depth to the water table, and distance from residential and industrial areas were significant factors affecting groundwater quality. Studies by Ngasala et al. [25] in Dar es Salaam, Tanzania, equally reported no correlation between well age and groundwater quality. In this study, there was a strong negative correlation between the depth of the well and the GWQI indicating that shallow wells were poorer in quality. This is similar to other areas such as Fokoslum, Ibadan, Southwest Nigeria, where Ahaneku [58] found that water quality was affected in shallow wells located up to 19.75m from pit latrines, and Marondera district, Zimbabwe, where Dzwairo et al. [59] assessed the impact of pit latrines on groundwater quality up to 25m. Field research showed that the majority of shallow wells were not protected from wastewater contamination, especially in shanty towns where it is common practice to empty pit latrines during floods. The strong correlation between distance and well depth indicate that the risk of poor groundwater quality increases with decreasing well depth. The strong correlation between the transmissivity of the aquifer formation and well depth also suggest that contamination decreases with increasing well depth.

The linear regression analysis shows that there is a relationship between the GWQI and the main parameters affecting the wells: the distance between the well and the nearest pit latrine, the depth of the well and the transmissivity of the aquifer formation. The results strongly explain the relationship between the GWQI and the significant parameters affecting the wells, as they confirm the correlation analysis (p ≤ 0.050). Our results clearly show how poor sanitation practices contribute to poor groundwater quality.

Most of the wells sampled in this study are private wells that supply water to the surrounding population. In addition, hydraulic properties such as the transmissivity of the aquifer formation, the discontinuity of the clay/sand/clay aquifer and the wells depth can significantly affect pollution levels. Ngasala et al. [25] drew similar conclusions from their study in Bangladesh, where they concluded that the minimum safe distance between a well and a pit latrine is a function of the hydrogeological conditions and the horizontal and vertical distances between the well and the latrine. Some studies have shown that certain types of soil can be used to filter and decontaminate water during infiltration. In a study conducted in Lichinga, Mozambique, Chaúque et al. [27] found that clay soil is a medium that appears to attenuate high levels of contamination in wells. In our study area, which is also located in a clay/sandy clay aquifer, the average distance measured between well and latrines is 53.60 m, with 32 % of wells located less than 15 m away. In addition, as these wells are used to supply water to the population, it is likely that the soil type acts effectively as a filtering medium. Our results are corroborated by the work of Ngasala et al. [60] who showed that groundwater contamination can be detected up to 13.4 m from the pit latrines, even in soil types that can act as filter environment.

The sanitary boundary defined in our study is consistent with the results of previous studies by Djousse et al. [28] who obtained a minimum distance of 42.89 ± 16.76m between sanitation facilities and water intake points in the Melen slum in Yaounde. Moreover, Strelkov et al. [[61], [62], [63]] classified sanitary protection zones into five different classes according to the location of existing water supply and sewerage facilities: zone I (1000 m), zone II (500 m), zone III (300 m), zone IV (100 m) and zone V (0 m).

## 5 Conclusion

Our results show that the poor quality of well water in the study area is due to the low depth of the wells and proximity of pit latrines to wells. The problem is exacerbated by the nature of the soil, which is described by the transmissivity of the aquifer formation. Well age and elevation do not appear to affect groundwater quality, but transmissivity of the aquifer formation and well depth have a very significant impact on groundwater quality, especially for shallow wells, as most shallow wells are very close to pit latrines. Detailed statistical analyses show a clear relationship between the significant factors affecting the wells and the groundwater quality index. Based on the results of our analyses, we propose a map of the sanitary boundary between well and pit latrines in our study area, which varies between 39 m and 370 m, with an average value of 215 m. This methodology can be applied to other similar study areas to protect and improve groundwater quality.

## Data availability statement

Data associated with the study is not deposited into a special public repository. However, it is included in the manuscript.

## CRediT authorship contribution statement

**William Teikeu A:** He is the one who wrote the first draft of the manuscript, he also proposed the méthodology and participated in field survey.**Visiy Edna Buhnyuy:** Data curation. **Kasi Njeudjang:** Writing – review & editing, Writing – original draft, Validation, Conceptualization. **Stephane Patrick Assembe:** Resources, Methodology. **Zakari Aretouyap:** Investigation, Formal analysis, Data curation. **Philippe Njandjock Nouck:** Validation, Supervision.

## Declaration of competing interest

The authors declare that they have no known competing financial interests or personal relationships that could have appeared to influence the work reported in this paper.
